# Phytochemicals in Breast Cancer Prevention and Therapy: Mechanisms, Efficacy, and Future Prospects

**DOI:** 10.3390/cimb47070527

**Published:** 2025-07-08

**Authors:** Neha Kodali, Chikezie O. Madu, Yi Lu

**Affiliations:** 1 Department of Biology, University of Memphis, Memphis, TN 38152, USA; nehakk2008@gmail.com; 2 Department of Biological Sciences, University of Memphis, Memphis, TN 38152, USA; comadu@memphis.edu; 3 Department of Pathology and Laboratory Medicine, University of Tennessee Health Science Center, Cancer Research Building, 19 South Manassas Street, Memphis, TN 38163, USA

**Keywords:** breast cancer, phytochemicals, prevention, therapy

## Abstract

Breast cancer is one of the most common forms of cancer in women globally. Phytochemicals are naturally occurring compounds in plants that have been the focus of many research studies for their potential in cancer prevention and treatment. This review will explore the mechanisms certain phytochemicals use to interact with the cellular pathways involved in breast cancer development. Phytochemicals modulate various processes such as apoptosis, cell cycle regulation, angiogenesis, and metastasis to potentially combat breast cancer. This review will also examine different dietary sources of phytochemicals, the potential for integration of phytochemicals into breast cancer therapy, the safety, toxicity, and limitations of phytochemicals, and the future of phytochemicals in the context of breast cancer.

## 1. Introduction

Breast cancer is a type of cancer that results in the largest number of annual deaths in women globally [1]. It makes up around 11.6% of all cancer diagnoses, one in four cancer cases in women, and one in six cancer deaths in women worldwide [2]. It has several subtypes, each with different treatment strategies and prognoses. Common subtypes include HER2-positive breast cancer, hormone receptor-positive breast cancer, and triple-negative breast cancer (TNBC). While many current treatment options have proven to be effective in some cases, many issues remain, including drug resistance, side effects, and disease recurrence [3]. Therefore, there has been a significant amount of research directed towards examining how phytochemicals can be used in cancer prevention and therapy [4].

Phytochemicals are biologically active compounds found in plants and are essential for the plant’s immune system and responses [5]. Such phytochemicals can be categorized into several groups, such as polyphenols (such as flavonoids and phenolic acids), terpenoids, alkaloids, and organosulfur compounds [6]. These phytochemicals have shown many anticancer properties, such as intervening in certain molecular pathways involved in breast cancer development, inducing apoptosis, preventing metastasis, regulating inflammation, and more [7].

This review will provide a comprehensive overview of the role of phytochemicals in breast cancer prevention and therapy. Categories and sources of relevant phytochemicals, the molecular mechanisms behind their anti-cancer effects, current epidemiological evidence, integration into existing treatments, safety considerations, limitations, and future directions will be discussed.

## 2. Classification of Phytochemicals Utilized in Breast Cancer Treatment

**Figure 1:**
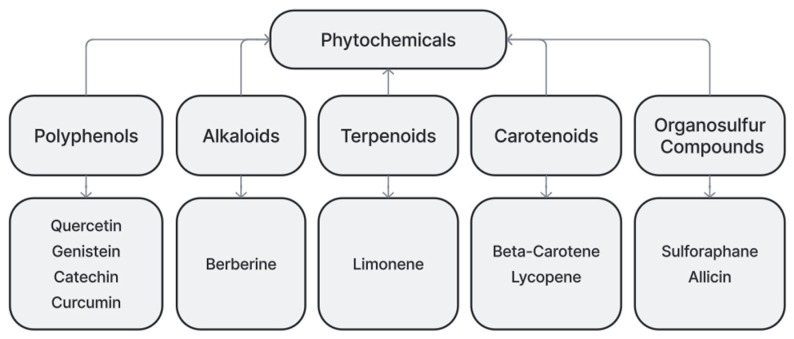
Diagram of example categorization of phytochemicals and examples.

Phytochemicals can be categorized into several different subgroups (see Figure 1).

Polyphenols are one of the more regularly studied classes of phytochemicals because of their antioxidant and anti-inflammatory characteristics [8]. They are able to prevent and hinder breast cancer progression through apoptosis, oxidative stress, angiogenesis, and cell cycle regulation [8].

Flavonoids are a type of polyphenol that are found in many fruits, vegetables, and teas [9]. Some significant types of flavonoids that have anti-breast cancer properties include quercetin, catechins, and genistein [10]. Quercetin, which can be found in apples, onions, and berries, has pro-apoptotic and anti-proliferative effects [11]. Catechins, which are found in green tea, have antioxidant properties and can inhibit breast cancer cell growth and metastasis [12]. Genistein, which is found in soybeans, is able to modulate estrogen receptors, making it effective for hormone-dependent breast cancer [13].

Phenolic acids are another type of polyphenol that have significant antioxidant and anti-inflammatory characteristics [14]. Ellagic acid, which can be found in pomegranates, several berries, and walnuts, is capable of performing cell cycle arrest and apoptosis in breast cancer cells [15]. Ferulic acid, which can be found in whole grains and seeds, has properties that protect DNA from oxidative damage and therefore inhibit the development of breast cancer [16].

Curcumin, which is found in turmeric, is a polyphenol in the diarylheptanoid class that has anti-inflammatory and apoptotic properties [17].

Carotenoids are fat-soluble pigments that have antioxidant and cell-signaling pathway modification properties [18]. Beta carotene, which can be found in carrots and sweet potatoes, is able to regulate apoptosis, which has led to it being linked to a reduced risk of breast cancer [19]. Lycopene, which can be found in tomatoes and watermelon, has been shown to inhibit cancer cell development and reduce oxidative stress [20].

Alkaloids are compounds that have nitrogen and have displayed anticancer properties [20]. Berberine, which is a compound found in all barberry species, has been shown to perform apoptosis and inhibit metastasis [21].

Terpenes and terpenoids are compounds that have antioxidant and anti-inflammatory properties [22]. Limonene, a terpene found in citrus fruits, has shown the ability to inhibit breast cancer development and induce apoptosis [23].

Organosulfur compounds are a class of phytochemicals that have strong antioxidant and anticancer properties [24]. Sulforaphane, which is found in broccoli sprouts, can induce apoptosis and inhibit breast cancer stem cells [24]. Allicin, which is found in garlic, can increase breast cancer cell death to decrease tumor growth and disrupt cancer cell signaling pathways [24].

## 3. Mechanisms of Action for Phytochemicals in Breast Cancer

**Figure 2:**
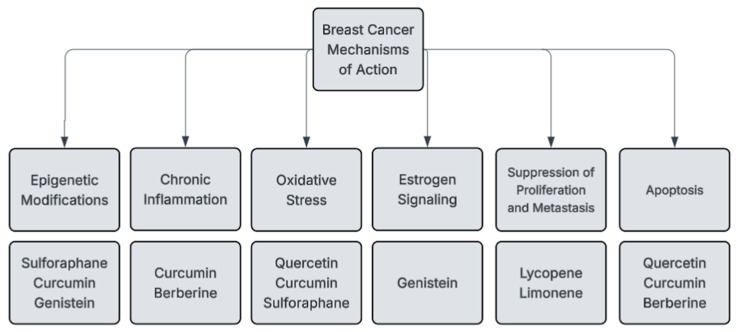
Example phytochemical anticancer pathways.

As previously mentioned, phytochemicals have several mechanisms for preventing breast cancer development and growth (see Figure 2 and Table 1). These mechanisms include antioxidant activity, estrogen signaling modification, apoptosis induction, proliferation inhibition, metastasis inhibition, inflammation suppression, and epigenetic modifications.

Oxidative stress is caused by an imbalance of ROS, or reactive oxygen species, and antioxidant defense [41]. This results in DNA damage, mutations, and irregular cell signaling, which all contribute to breast cancer progression [42]. Quercetin, the flavonoid, seeks out ROS, increases antioxidant enzyme activity to neutralize ROS, and protects DNA from oxidative damage [25]. Curcumin, the polyphenol, suppresses lipid peroxidation and activates the Nrf2 pathway, which regulates genes responsible for antioxidant responses [29]. Sulforaphane, the organosulfur compound, triggers phase II detoxification enzymes, which assist with cellular antioxidant defenses [38].

Many breast cancers depend on estrogen signaling for development, especially estrogen-receptor types. Some phytochemicals are considered phytoestrogens, estrogen pathway modulators that can hinder the development of such breast cancers [43]. Genistein, the isoflavone, binds to estrogen receptors ERα and ERβ, resulting in both estrogenic and anti-estrogenic effects, which can reduce the risk of breast cancer [13].

Apoptosis, or intentional cell death, is a natural defense mechanism against cancer that phytochemicals are able to induce by activating pro-apoptotic proteins and inhibiting survival pathways [44]. Quercetin inhibits PI3K/Akt signaling, which results in increased Bax, a pro-apoptotic protein, and decreased Bcl-2, an anti-apoptotic protein, levels [26,27]. Curcumin activates caspase-3 and caspase-9, which are important enzymes in apoptosis induction [30,31]. Berberine, the alkaloid, can induce apoptosis through mitochondrial dysfunction and activation of the p53 tumor suppressor pathway [35].

Phytochemicals can suppress proliferation and metastasis, the ability of the cancer to spread, by targeting certain signaling pathways [34]. Lycopene, the carotenoid, is able to decrease cyclin and CDK production and activity to prevent uncontrollable growth [34]. Limonene, the monoterpene, is able to disrupt Ras signaling, which is necessary for tumor proliferation [37]. Sulforaphane is able to suppress epithelial–mesenchymal transition, or EMT, which is an important process in metastasis [39]. Chronic inflammation creates an environment that supports tumors and therefore promotes breast cancer [45]. Many phytochemicals have anti-inflammatory and immune-regulating characteristics [46]. Curcumin inhibits NF-κB, which is a transcription factor that regulates inflammatory cytokines, such as IL-6 and TNF-α [32]. Berberine reduces macrophage-induced inflammation in the tumor microenvironment [36].

Epigenetic modifications, such as DNA methylation and histone modifications, can regulate gene expression by modifying the DNA sequence [47]. Cancer cells can undergo epigenetic changes that promote tumor growth, and certain phytochemicals can reverse such changes and restore normal gene function [48]. Sulforaphane inhibits histone deacetylases, or HDACs, which leads to the reactivation of tumor-suppressor genes [40]. Curcumin modifies DNA methylation patterns and is able to restore normal gene expression [33]. Genistein affects miRNA expression, which regulates cancer-related genes [28].

## 4. Phytochemicals and Breast Cancer Stem Cells

Breast cancer stem cells (BCSCs) are a subpopulation within tumors that are capable of self-renewal, differentiation, and tumor growth initiation [49]. They have a natural resistance to chemotherapy and radiation therapy, and this significantly contributes to treatment failure, metastasis, and recurrence [49]. Therefore, treatments that can target BCSCs are promising for the improvement of clinical outcomes of breast cancer patients. Recent research has shown that certain phytochemicals can modify the survival and function of BCSCs [49].

Curcumin, the polyphenol, has shown such effects on BCSC populations by suppressing the Wnt/β-catenin pathway, a primary regulator of stemness [50]. Curcumin has also been shown to downregulate aldehyde dehydrogenase 1 (ALDH1) expression, which is a marker often associated with BCSC enrichment [50]. Sulforaphane, the organosulfur compound, has been shown to inhibit mammosphere formation and decrease populations of CD44^+^/CD24^−^ cells, markers that indicate BCSCs [51,52]. Sulforaphane also sensitizes the cells to conventional therapies, making them more effective [52]. Genistein, the isoflavone, attacks epigenetic regulators of stemness and has been found to decrease BCSC activity through modulation of microRNAs involved in the expression of differentiation pathways [53]. Other compounds, like resveratrol and epigallocatechin gallate (EGCG), have shown similar effects by inhibiting self-renewal signaling networks [51].

The preclinical data appears extremely promising. However, integration into clinical practice remains a challenge as the issues of bioavailability, dosage, and potential toxicity at higher concentrations are yet to be addressed. Nonetheless, the targeting of BCSCs with phytochemicals still seems to be a compelling treatment option in the future if these problems are to be resolved.

## 5. Phytochemicals and Immune Modulation in Breast Cancer

The immune system plays a key role in controlling tumor growth. Therefore, immunomodulation has been gaining more focus in cancer therapy. Phytochemicals have been proven to be a possible method of increasing anti-tumor immunity while alleviating immune evasion strategies used by cancer cells.

Many phytochemicals have been shown to initiate innate and adaptive immune responses. Resveratrol improves the cytotoxic activity of CD8^+^ T lymphocytes and natural killer (NK) cells [54,55]. It does this while also simultaneously inhibiting the proliferation of regulatory T cells (Tregs) that suppress anti-tumor immunity [56]. Curcumin is able to decrease levels of pro-inflammatory cytokines such as interleukin-6 (IL-6) and tumor necrosis factor-alpha (TNF-α) in order to control the tumor immune microenvironment [32]. It does this while also upregulating anti-inflammatory cytokines like interleukin-10 (IL-10) [57].

Flavonoids, like quercetin and apigenin, have displayed the ability to downregulate immune checkpoint molecules, such as programmed death-ligand 1 (PD-L1) [58]. As a result, it sensitizes tumors to immune checkpoint inhibitors [58]. Sulforaphane has been shown to increase dendritic cell maturation and antigen presentation, which are important steps in the activation of effective adaptive immune responses against tumors [59].

Phytochemicals may also alter the composition and function of the gut microbiota, which has an important role in regulating systemic immune responses and therapeutic results in cancer [60]. For example, polyphenols are capable of increasing the amount of helpful microbial species that create short-chain fatty acids, which have immunoregulatory properties [61,62].

Phytochemicals’ immunomodulatory potential encourages their integration into combination therapies with conventional treatments. However, it is key to maintain careful dosing and scheduling to avoid unintended immunosuppression. Extensive research is necessary to refine these combinations, as well as to clarify the long-term impacts of phytochemical-induced immune modulation.

## 6. Clinical Trials and Human Studies on Phytochemicals in Breast Cancer

Despite several in vitro and in vivo experiments that support the anticancer potential of phytochemicals, the transition into clinical studies has been slow due to the problems that remain, such as study design, bioavailability, and regulatory issues. Regardless, there have been several clinical trials that have explored and provided positive data on the use of phytochemicals in breast cancer prevention and treatment.

Curcumin, one of the more regularly studied phytochemicals, has been the subject of several clinical experiments. Curcumin was found to be safe and well-tolerated at doses up to 8 g per day in a phase I clinical trial [63]. However, systematic absorption issues were noted. Other studies have studied curcumin as a complement to chemotherapy. In such studies, it has been found to improve the tolerability of treatment and reductions in inflammatory biomarkers like NF-κB and COX-2 [64,65].

Green tea catechins, mainly EGCG, have been extensively studied in clinical studies. One randomized controlled trial involved women with early-stage breast cancer and showed that green tea supplementation was associated with reduced proliferation indices in breast tissue [66]. This displayed the potential chemopreventive effects of green tea catechins. However, there have been inconsistencies in dosing, participant characteristics, and treatment duration in such trials, and these inconsistencies have prevented definitive conclusions.

## 7. Challenges in Phytochemical Research Methodology

Research into phytochemicals and their anticancer effects has been met with several methodological challenges that have complicated the interpretation and translation of findings. One prominent issue is the difference between concentrations deemed effective in vitro and achievable systemic levels in vivo [67]. Phytochemicals often display anticancer effects in cell culture models at micromolar concentrations [68]. However, they may only reach nanomolar levels in human plasma following oral ingestion [69].

Phytochemicals also go through extensive metabolism in the liver and gut, which produces several metabolites with unique biological activities [70]. As a result, several studies only focus on the parent compounds and may not take contributions from these metabolites into account [71]. Additionally, the bioavailability and pharmacokinetics of phytochemicals can vary to a large extent [72]. This is dependent on the formulation, dietary content, and individual patient factors such as microbiome composition [73].

Another issue is experiments using oversimplified experimental models. Traditional two-dimensional (2D) cell cultures do not accurately depict the complex three-dimensional (3D) makeup of tumors or the tumor microenvironment [74]. This includes the numerous interactions with immune cells, fibroblasts, and extracellular matrix components [75]. 3D spheroids, organoids, patient-derived xenografts (PDXs), and other advanced models provide more physiologically relevant systems for studying the anticancer effects of phytochemicals [76].

Another challenge is that phytochemicals often display pleiotropic effects, meaning they act on multiple molecular targets simultaneously [77]. While this property can be beneficial in overcoming resistance mechanisms, it complicates the ability to interpret precise mechanisms of action. High-throughput omics technologies, such as transcriptomics, proteomics, and metabolomics, are being used more to determine the complex networks created by phytochemicals [78].

Finally, extraction methods, purity levels, and experimental protocols have shown variability across studies, and this has further complicated cross-comparisons and meta-analyses [79]. Standardization in research practices, transparency in reporting, and adherence to rigorous experimental design will all be necessary to advance phytochemical research.

## 8. Potential for Integration into Breast Cancer Therapy

Phytochemicals have shown many anticancer characteristics, making them promising for breast cancer treatment and prevention. However, this faces several issues, such as bioavailability, drug interactions, and standardization [80,81,82].

While current breast cancer treatments are effective, they can often lead to side effects, drug resistance, and tumor recurrence [83,84]. Many chemotherapeutics cause oxidative stress, inflammation, and immune suppression, which results in toxicity in normal tissues [82,83]. Phytochemicals can overcome these issues by improving the body’s antioxidant defenses and reducing inflammation [85].

Many cancer cells can develop mechanisms that allow them to resist the effects of chemotherapy. Phytochemicals can help with this by interfering with resistance pathways, improving drug sensitivity, and increasing drug uptake by cancer cells [86]. They can also disrupt multiple molecular targets at the same time, making it difficult for cancer cells to adapt and survive [87].

Despite these benefits, bioavailability is an issue that prevents phytochemicals from being widely used clinically [80]. This means that they break down too quickly or are excreted before their effects can occur, limiting how effective they can be [80]. Innovations in drug delivery systems, however, may be able to improve absorption, stability, and controlled release of phytochemicals within the body [80].

## 9. Safety, Toxicity, and Limitations

The therapeutic use of phytochemicals would inevitably require considerations of safety, potential toxicity, drug interactions, and regulatory challenges.

High doses of phytochemicals can lead to adverse effects, as some compounds lead to hormonal activity, cytotoxicity, or pro-oxidant effects when they are consumed excessively [88]. Excess consumption can also lead to metabolic pathway overload, which can result in liver toxicity, gastrointestinal distress, or immune suppression [89]. Therefore, further clinical research will be needed to establish safe dosage thresholds.

Phytochemicals can also alter the metabolism of chemotherapy drugs, hormone therapies, and immunotherapies [90]. Since they often can influence enzyme activity, specifically those involved in drug metabolism, phytochemicals can either increase or decrease drug effectiveness [90]. This can result in failed treatments or an increase in toxicity [90]. Since phytochemicals can also affect cell signaling pathways, anticancer drugs may also face decreased effectiveness [90].

## 10. Future Prospects and Emerging Research

As the understanding of phytochemicals and their potential integration into treatments for breast cancer deepens, there are many prospects for this integration.

One idea that has been researched is personalized phytotherapy [91,92]. This would involve creating specific phytochemical treatments for a patient based on their genetic profile, tumor type, and metabolic properties [91,92]. Genomic and transcriptomic data would allow researchers to identify biomarkers that would predict responsiveness to various phytochemicals [91,92].

Artificial intelligence and computational biology are also contributing to phytochemical research [93]. Such technologies are able to search large sets of data regarding phytochemicals’ structure and function and use this data to predict their anticancer potential [93]. This would significantly increase the rate of discovery for phytochemicals in the context of breast cancer, as well as the rate of drug design [94]. Network pharmacology may be used in parallel to determine complex interactions between phytochemicals and cellular signaling pathways to provide a deeper understanding of the many effects of such phytochemicals [95].

Advanced drug delivery systems, as previously mentioned, are also being researched [96]. Because of poor bioavailability of phytochemicals, nanoparticle carriers, liposomal encapsulation, and biodegradable hydrogels are currently being investigated [96,97]. Such technologies would both increase the effectiveness of treatments and reduce toxicity [96].

## 11. Ethical and Regulatory Perspectives on Phytochemical Use

The increased availability, marketing, and popularity of phytochemical supplements and functional foods for cancer prevention and therapy have raised important ethical and regulatory concerns. Pharmaceutical drugs are heavily regulated, but many phytochemical products are regulated as dietary supplements [98]. This means that they are often put under less rigorous standards for safety, efficacy, and marketing claims [98].

One ethical issue is consumer misinformation. Phytochemical products have often been marketed with claims regarding cancer prevention or treatment that lack substantial evidence [99]. This could potentially lead to patients not using evidence-based therapies. Vulnerable populations, such as those with limited healthcare access, may be particularly susceptible to misleading promotions and marketing. Regulatory agencies like the U.S. Food and Drug Administration (FDA) and the European Medicines Agency (EMA) have set guidelines to minimize false health claims [100,101]. However, enforcement of such guidelines may be inconsistent. Additionally, the lack of standardized manufacturing methods results in variability in product quality. This may result in some supplements having lower concentrations of active ingredients than advertised or being contaminated with harmful substances.

Another ethical concern is how phytochemical research itself is conducted. Clinical trials must make sure of informed consent, especially taking into account the experimental nature of phytochemical interventions and the lack of definitive efficacy data. Trial designs should have appropriate control groups and endpoints to provide patients and clinicians with useful and worthwhile information.

There is a need for stronger regulations regarding the production, marketing, and clinical evaluation of phytochemical products for these challenges to be addressed. Ethical marketing practices, improved product labeling, and transparent communication about the benefits and limitations of phytochemicals are necessary to protect consumers and encourage evidence-based decision-making.

## 12. Perspectives from Traditional Medicine

Traditional medicinal practices, such as Traditional Chinese Medicine (TCM), Ayurveda, and indigenous healing practices, have historically used phytochemicals in the prevention and treatment of breast disorders, including malignancies [102]. These practices use a holistic approach to health and perceive diseases as disruptions of a systemic balance instead of a localized pathology.

In TCM, breast cancer has been historically classified as “breast lumps” or “breast carbuncles,” and it is often treated with herbal formulas that clear “heat,” resolve “phlegm,” and revive blood circulation [103]. Herbs such as *Scutellaria baicalensis* (containing baicalein) and *Hedyotis diffusa* (high in saponins and flavonoids) have shown anticancer characteristics in modern studies [104]. Ayurveda also uses phytochemical-heavy plants such as *Withania somnifera* (ashwagandha) and *Curcuma longa* (turmeric) to improve immune resilience and suppress tumor growth [105]. Both are now extensively studied for their capabilities of modulating inflammation, apoptosis, and oxidative stress.

Indigenous medical practices were often transmitted orally and adapted over generations. These practices also used local plants with anticancer potential. For example, indigenous North American communities have historically used plants such as *Podophyllum peltatum* (mayapple), where the chemotherapeutic agent podophyllotoxin comes from [106]. Similarly, indigenous people in Peru have used the Amazonian *Uncaria tomentosa* (cat’s claw) for its anti-inflammatory and immune-modulating characteristics [107].

These traditional practices often use synergistic herbal combinations, and this may improve bioavailability and therapeutic efficacy compared to using isolated compounds. Modern research is continuously validating these practices, providing new pathways for drug discovery. However, complete integration of traditional medicine into contemporary clinical settings requires extreme scientific validation and support. Key steps include the standardization of herbal preparations, identification of active components, and clinical trials that evaluate efficacy and safety. Traditional knowledge must also be respected and protected from biopiracy and misappropriation.

## 13. Conclusions

Phytochemicals have shown several anticancer characteristics, which are promising for working alongside current breast cancer treatments to improve treatment efficacy and decrease side effects. However, they still face certain issues, including bioavailability, drug interactions, and standardization, which must be resolved before they can be widely accepted.

## Figures and Tables

**Table 1:** Table of phytochemicals, natural sources, and observed effects.

Phytochemical	Natural Source	Observed Effects	References
Quercetin	Apples, onions, berries	Increases antioxidant enzyme activity to neutralize ROS, protects DNA from oxidative damage, inhibits PI3K/Akt signaling	[25,26,27]
Genistein	Soybeans	Binds to estrogen receptors ERα and Erβ, affects miRNA expression	[13,28]
Curcumin	Turmeric	Suppresses lipid peroxidation and activates the Nrf2 pathway, activates caspase-3 and caspase-9, Inhibits NF-κB for anti-inflammatory effects, modifies DNA methylation patterns to restore normal gene expression	[29,30,31,32,33]
Lycopene	Tomatoes, watermelon	Decreases cyclin and CDK production and activity to prevent uncontrollable growth	[34]
Berberine	Barberries	Induces apoptosis through mitochondrial dysfunction and activation of the p53 tumor suppressor pathway, Reduces macrophage-induced inflammation in the tumor microenvironment	[35,36]
Limonene	Citrus fruits	Disrupts Ras signaling, which is necessary for tumor proliferation	[37]
Sulforaphane	Broccoli sprouts	Triggers phase II detoxification enzymes, Suppress epithelial–mesenchymal transition to prevent metastasis, Inhibits histone deacetylases for reactivation of tumor-suppressor genes	[38,39,40]
